# Physicians Interrupted by Mobile Devices in Hospitals: Understanding the Interaction Between Devices, Roles, and Duties

**DOI:** 10.2196/jmir.2473

**Published:** 2013-03-07

**Authors:** Terje Solvoll, Jeremiah Scholl, Gunnar Hartvigsen

**Affiliations:** ^1^University Hospital of North NorwayNorwegian Centre for Integrated Care and TelemedicineTromsøNorway; ^2^University of TromsøDepartment of Computer ScienceTromsøNorway; ^3^Karolinska InstitutetHealth Informatics CentreJämtlands LänSweden

**Keywords:** Ethnography, Mobile communication, interruptions, Context-aware computing, Pervasive computing, User-centered design methods, Health care, Pagers, Wireless phones, CSCW, HCI

## Abstract

**Background:**

A common denominator of modern hospitals is a variety of communication problems. In particular, interruptions from mobile communication devices are a cause of great concern for many physicians.

**Objective:**

To characterize how interruptions from mobile devices disturb physicians in their daily work. The gathered knowledge will be subsequently used as input for the design and development of a context-sensitive communication system for mobile communications suitable for hospitals.

**Methods:**

This study adheres to an ethnographic and interpretive field research approach. The data gathering consisted of participant observations, non-structured and mostly ad hoc interviews, and open-ended discussions with a selected group of physicians. Eleven physicians were observed for a total of 135 hours during May and June 2009.

**Results:**

The study demonstrates to what degree physicians are interrupted by mobile devices in their daily work and in which situations they are interrupted, such as surgery, examinations, and during patients/relatives high-importance level conversations. The participants in the study expected, and also indicated, that wireless phones probably led to more interruptions immediately after their introduction in a clinic, when compared to a pager, but this changed after a short while. The unpleasant feeling experienced by the caller when interrupting someone by calling them differs compared to sending a page message, which leaves it up to the receiver when to return the call.

**Conclusions:**

Mobile devices, which frequently interrupt physicians in hospitals, are a problem for both physicians and patients. The results from this study contribute to knowledge being used as input for designing and developing a prototype for a context-sensitive communication system for mobile communication suitable for hospitals. We combined these findings with results from earlier studies and also involved actual users to develop the prototype, CallMeSmart. This system intends to reduce such interruptions and at the same time minimize the number of communication devices needed per user.

## Introduction

The work setting in hospitals is communication intensive and can lead to significant difficulties related to interruptions from other co-workers [1-4]. One suggested solution for this problem is to implement wireless phone systems [5-7]. Such systems offer a number of advantages over traditional paging systems, such as not requiring the staff to find a phone after being paged. Moving from wired phones to wireless communication platforms has a significant impact in the future of health care delivery systems [8]. Mobile phones’ interference with electronic equipment is, according to [9], not an issue anymore, or it can be solved by the solutions in [10].

The adoption of wireless phones, however, does not come without risk. Psychological theory [11] and empirical evidence [12] both suggest that wireless phones have the potential to create additional problems related to interruptions, such as conversations and questions that would not normally occur, when compared to using traditional paging systems. This has caused some hospital staff to resist the adoption of wireless phone systems when given the opportunity [13]. Further studies regarding the nature and effects of interruptions from wireless phone systems in hospitals have thus been suggested [14].

There have been many suggestions on how to reduce interruptions from mobile devices over the years. Many of these systems are based on using contextual information to reduce interruptions. Some of these systems change the configuration of the phone automatically [15-18] and includes quiet calls where the receiver could negotiate with the caller through text or pre-recorded audio messages. However, this negotiation does not reduce personal interruptions in that the user must still act when receiving a call. Other systems focus on giving the caller information about the receivers’ context and thereby helping the caller make decisions on when to call [19-21]. Avrahami et al discovered that if they provided the caller with contextual information about the receiver’s situation, it reduced the mismatch between the caller’s decision and the receiver’s desires [22]. To our knowledge, none of these systems has been tested in hospital settings.

Hospitals are dependent on a wide and reliable communication infrastructure for exchanging different kinds of data, including text, voice, and alarm services. Several studies aiming to improve communication and reduce interruptions have been carried out, also within hospital settings, without major success [3,5-7,23,24]. Personal Digital Assistants (PDAs) have also been tested in a contextual message exchange system without major success [25]. Other systems, like the AwareMedia and AwarePhone systems of Bardram et al [26,27], support context-sensitive communication and form a complete communication system for clinicians in a surgical ward. The AwarePhone system is an application running on a mobile phone (GSM/3G) that is not integrated in the hospital’s internal communication infrastructure, therefore, it requires the user to carry another device. The feedback from its users focused on privacy issues as one of the major drawbacks.

In this paper, we present an interpretive case study regarding interruptions from mobile devices at a large hospital in Norway, which started implementing a pervasive wireless phone system in 2006. This setting offers the opportunity to gain perspective from health staff with several years of experience using wireless phone systems. The goal of the study was to characterize a physician’s workday, focusing on wireless communication. The aim was to understand the health care workers’ communication pattern and how unnecessary interruptions from mobile devices can be reduced: in which situations, what context, and which location. Such design-oriented studies using methods from computer-supported cooperative work have been suggested for medical informatics systems in general [28], and medical collaboration systems in particular [29], in order to improve their overall effectiveness and success rate upon implementation. This knowledge contributes to medical informatics by improving understanding of the effects of the use of wireless phone systems and is used as input to the overall sociotechnical development of a wireless communication system for hospitals.

## Methods

### Research Setting

The study was conducted at St. Olavs Hospital, Trondheim University Hospital, Norway, which is a health enterprise in Mid-Norway health region that consists of 695,000 inhabitants in total. St. Olavs Hospital is under renovation, and the hospital project consists of the construction of new buildings and a new hospital organization. The first new clinical centers were completed in 2006, and the entire project will be completed in 2015. The new St. Olavs hospital is a technologically advanced hospital, and the communication system is one of the world’s most modern hospital communication systems. The existing system is based on wired and wireless (Internet Protocol) IP-phones from Cisco. The wireless IP-phones are the technology of interest for this research. The model in use at the studied hospital is the Cisco 7921G wireless phones. They also use an old paging system, both for on-call duty pagers and personal pagers, and a GSM-based on-call duty phone in parallel as back-up, since the older parts of the hospital do not have coverage for wireless IP-phones. The pagers were from different vendors but were all of the simple type that can receive only the number from the caller. The GSM-phone was an ordinary Nokia mobile phone. Most of the physicians also carried a private mobile phone.

Two separate clinics were included in the study: the Ear, Nose, and Throat (ENT) Clinic and the Child and Youth (CY) Clinic. Two clinics from the same hospital were included in order to provide a broader understanding on the usage and experiences of wireless phones, compared to studying only one clinic. This also provided us the opportunity to compare and contrast the data collected between the separate clinics.

### Research Method

The study adheres to an ethnographic and interpretive field research approach [30-33]. Ethnography and participant observations represent a uniquely humanistic interpretive approach [33]. Interpretive research has the potential to explain the human thought and action in a social and organizational context [31]. Principles for trusted pervasive health have also been under consideration [34,35].

Data gathering conducted by the first author consists of participatory observations, nonstructured and mostly ad-hoc interviews, and open-ended discussions of a selected group of physicians at various levels of hierarchy and roles, within two clinics at St. Olavs Hospital. The participatory observer (hereafter referred to simply as “observer”) was from a different institution at a different location than the hospital where the study was performed. The observer’s background is computer science, communication, and medical informatics, and he was not medically trained to perform this study. This was fully understood by the participants, who were also aware that the observer was a researcher. The observer signed a nondisclosure agreement regarding sensitive information prior to the observations.

#### Participant Observations

The fact that peoples’ descriptions of their activities often differ from what really occurs in practice is one of the main rationales for doing observations. There can be several reasons for this phenomenon, such as the limitation of the human memory, people not always being aware of their actual behavior, peoples’ concern with their image (and therefore giving a better story than the real one), and also the complexity of social life causing different people to report differently.

The observer followed the independent work of 11 physicians, including 2 cancer teams and 2 surgical teams at two clinics, for a total of 135 hours during May and June 2009. The purpose of the work was to observe the daily work of physicians and to interview them, focusing on interruptions from mobile devices. The observer stayed at the ENT clinic for a total of 65 hours: 26 hours observing assistant physicians and 39 hours observing chief physicians. At the CY clinic, he stayed for a total of 70 hours. 15 hours were used to observe assistant physicians, 47 hours observing chief physicians, and 8 hours to observe one chief and one assistant physician working on a cancer team. The observer took the role of a first-year medical student, dressing and acting like a physician to blend in as much as possible for a more realistic picture of the communication situation at the clinic. This technique is often referred to as “shadowing” [36]. He followed each physician in their everyday work at outpatient clinic, surgery, cancer meetings, etc, for at least 2 workdays/nights/duties. The head of the clinic chose physicians and roles to form an average representation of the physicians and roles at the clinic. The observer had to contact each physician to make an appointment for each observation, which was done during the morning meeting at each clinic. There were some changes of the selected physicians and roles due to shifts, which resulted in increased observing hours and did not influence the representativeness of the clinic. The observer registered every call/page/message, type of device, reaction, and context for each physician. Depending on the situation, he also asked questions related to the context and the communication device used.

Data were recorded using pen and paper on a self-constructed four splitter form, registering: situation, device, time (start - stop), and reaction/response (meaning, answer or not, by whom, what context, etc). The reaction/response was recorded as free text notes. The questions made by the observer were related to the context and the communication device (if any). They were asked directly after the event, and the answers written down in free text notes on the form. We do not believe this had any effect on the subjects’ behavior and the generalization of the results, due to the nature of the questions: “Was this a call related to your professional role on duty or role-independent personal call?”, “Was this considered as an important call that you had to answer, or was it a more general question that another physician could have answered?”, etc. In most of the cases, the subject informed the observer unsolicited. The decision about using pen and paper instead of a digital device such as a PDA, as used in [37-40], was made since these physicians did not use these kinds of devices in their daily work. This helped the observer blend in as much as possible in the health care workers settings and avoided unintended attention paid by the workers to the observations.

#### Interviews/Discussions

Interviewing is also an important method within ethnography. Interviews increase understanding of what has been observed and the subjects’ perspective of a specific situation. One way to interview is during an on-going activity like we did, during the observations. In this way, we connected the observations with the interview and therefore got answers to questions about the observed activity, which helped us understand the situation from the subjects’ perspective.

During the observation period at each clinic, the observer had office accommodation in an open-plan office among the assistant physicians. This created the opportunity for several discussions and also the collection of input on how to improve the communication system—mostly on improving the user experience, but also on how to reduce the interruptions. While observing the physicians, the observer was allowed to interview and talk to the physicians, but he remained quiet during patient consultations. All questions and discussions, both during observation and office time, were conducted in Norwegian and notes were taken from them, but not recorded. Interview guides were not used, but there were questions related to context and what was observed. Some of the questions were asked of all participants, while others were specific to the situation. The initial focus of the interviews and discussions was the use of wireless mobile communication devices regarding improvement of interruption management.

### Data Analysis

Data analysis was conducted concurrently with the data collection process using Grounded Theory. Initial data analysis began with the first author reviewing all the notes and reflecting on some general issues that seemed inherent to the data. It was clear at this point that, despite there being some similarities, there also were differences with respect to the two clinics. A decision was then made to analyze the data from the two clinics separately. For each section the data were analyzed around five basic themes: (1) frequency of interruptions, (2) the clinical situation, (3) context [41], which included location, (4) comparisons of pagers vs wireless/mobile phones, and (5) answered or not vs returned call after a page, and the importance of the call. Exploring these data was an adequate approach to gaining an understanding of the communication in hospitals. The data indicate the communications’ frequency, length, and importance, if they were interruptive or not, and the reaction from the user. Analyzing log data is a satisfactory approach to mapping out the actual communication patterns. It is also a useful approach to visualize the numbers and the length of communication sessions, and how often the users were interrupted unnecessarily. Analyzing observed situations and then comparing them with comments and answers during the interviews and discussions worked well for mapping the communication situation and capturing and understanding potential changes over time.

## Results

Although there were many similarities between the two clinics, there were also a number of differences regarding communication patterns and the handling of different situations. The results from the clinics are therefore presented separately.

A general similarity between the clinics was that each physician at both clinics carried at least one wireless IP-phone, one on-call duty pager, and one private mobile phone. Some of them, mostly assistant physicians, also carried a personal pager and, during evening and night duty, a backup GSM phone was also required. To use the wireless IP-phone, the physician had to personally log on to the phone, and if they also were responsible for a role, they had to log on to that role too. When referring to a physician, we will call them Phys-A to K, and nurses as Nurse-A to F.

### Ear, Nose, and Throat Clinic

The Ear, Nose, and Throat (ENT) clinic has a regional function for the Mid-Norway health region. The clinic treats problems that the general practitioners or private specialists in ear, nose, and throat, have not been able to treat. The clinic also incorporates jaw- and eye- units, but we concentrated our observations to the ENT-unit, which consisted of the following sections: outpatient clinic, surgery, and inpatient ward. The clinic takes care of work-up, diagnostic, medical, and surgical treatment within cancer, nose, sinus treatment, etc. We observed assistant physicians’ role as on-call duty at the inpatient ward and outpatient clinic, in surgery, and also observed the chief physician’s role as on-call duty at the inpatient ward, outpatient clinic, in surgery, and in cancer meetings. A cancer meeting was a team of physicians meeting/examining and treating cancer patients.

#### Frequency of Interruptions

One of the assistant physicians, Phys-A, said on the first day of observations: “It varies a lot how much we are interrupted from various mobile devices. The busiest time is definitely between 8 in the morning and 7-8 in the evening”. However, only a few hours that we observed at this clinic were considered as “normal” regarding interruptions. Table 1 shows all interruptions from mobile devices received by the participating physicians at this clinic.

During the observations the physicians and/or their nearest colleagues commented on the frequency of interruptions during the day. A chief physician, Phys-B, stated one day during outpatient clinic: “Unusually quiet on the phone today or actually it has been this quiet all week. It seems like many have started their vacation”, and a theatre nurse, Nurse-A, said during a surgery, “This has to be the first surgery I’ve performed together with NN where he has not been constantly interrupted by the phone”. During a cancer meeting, the chief physician, Phys-B, said, “This is a quiet day regarding calls, but it is publicly known at the clinic which days I have these meetings”, which was continued by the head nurse, Nurse-B: “The phone should not ring when we are in these kind of meetings. The rule is that a secretary answers the phone and takes messages”. Even though most of the observation hours at this clinic were considered as “quiet”, Table 1 show that they were interrupted several times in situations when they should not have been unnecessarily interrupted. Most of the interruptions were mentioned afterwards as “not important” and could have been postponed until the physician becomes available, or answered by others.

**Table 1:** Overview of total amount of interruptions from mobile devices at ENT during observation time (only interruptions from the followed physicians’ devices, except for *g*, which was a nurses’ phone brought to the physician).^a^

			Assistant physician	Chief physician
**Preparatory/complementary work**				
		Answered	3a	8a
		Ignored		
**Outpatient ward**				
	No patient			
		Answered	1b	3a
		Ignored		
	With patient			
		Answered	2a,8b,1c, 3e,1g	
		Ignored		
**Inpatient ward**				
	No patient			
		Answered		1a
		Ignored		
	With patient			
		Answered		
		Ignored		1a
**Surgical theatre**				
		Answered	1a	5a
		Ignored		1a
**Meeting**				
		Answered	3a	2a
		Ignored		
**Cancer meetings**				
		Answered		3a, 1b
		Ignored		1a, 2b
**Other situations**				
		Answered	1a, 1b	11a
		Ignored		

^a^a=wireless IP-phone; b=on-call-duty pager; c=backup on-call-duty GSM-phone; e=Wired IP-phone; g=other

While observing in the outpatient clinic, we discovered that the health care workers at the clinic often sought the physician in outpatient duty “in-person” instead of calling him/her. In this way, they grasped the context immediately and knew if they could interrupt or not. We also observed that the less experienced physicians often used the nurses to contact the experienced physicians, instead of calling them themselves. In this way, they could continue their own work while waiting for an answer. We observed such situations at the outpatient clinic, where normally a nurse came into the room and brought her own wireless IP-phone to the physician, and asked if he/she could answer a question from the caller.

Another situation that was observed was that calls/pages during meetings/lectures at the clinic normally were answered. This was also pointed out by the head of the clinic:

There are only two physicians that have on-call duty during each meeting/lecture, but regardless, everyone immediately answers incoming calls/pages… This is not necessary, and the most annoying part is when the person picks up the phone and starts to talk on their way out.

#### Interruptions During Surgery

Table 1 show that physicians at this clinic also responded to phone calls during surgery. In this case, rather than answering the phone themselves, somebody else in the room answered the call for them, normally a nurse or another physician. The person who answered the phone either took a message or held the phone up to the physician’s ear. After such an incident during a surgery where the theatre nurse, Nurse-B, answered the chief physician’s phone and held the phone up the physician’s ear, she said:

Normally we only take messages or convey the question to the physician, but, like now, if it is ok for the physician, we could hold the phone for the physician, which happens rarely. Most of the calls are questions that could be conveyed by the nurse and then answered through the nurse. Normally after a surgery, there are more or less 10 messages waiting for the physician that could be difficult to manage. Most of these calls have to be returned anyhow.

We also observed that the pager was left in the physician’s coat outside the surgical theatre, and they brought with them only their private mobile phone and the wireless IP-phone.

#### Wireless Phones vs Pagers

The physicians at the clinic appeared to be pleased with carrying a wireless IP-phone, and during discussions about carrying wireless phones in the new hospital compared with pagers, a chief physician, Phys-B, said:

People are generally satisfied with carrying phones compared to the old days when we only had pagers, but it took a while to adjust to the new system. After some time you learn to screen the calls, the important ones from those that are not so important. With a pager you never know, and you therefore have to return the call as soon as possible.

In response to the question about the phone being more interruptive compared to the pager-only situation, he said: “The phone is less interruptive compared to the pager. I do not know if it is me who has been better at telling people when I’m available and managing the communication, or if it is the others who have been better.” This also seemed to be the general view at this clinic. Another important point regarding why there may be fewer interruptions from the phone compared to when they had only pagers, was made by a chief physician, Phys-E: “If you call someone and they are busy, and your interruption is not important enough, you risk an unpleasant situation, while paging someone is not that risky…”.

### Child and Youth Clinic

The Child and Youth (CY) clinic treats patients aged between 0-16 years, mainly from the mid-Norway health region, but also from other parts of Norway. The clinic consists of 10 units, where we concentrated on the outpatient child and youth unit, cancer and blood diseases unit, and child and newborn intensive care units. We observed one general chief physician, one newborn intensive care chief physician, one chief physician in cancer and blood diseases, two assistant physicians, in two roles: primary watch duty and intermediate watch duty, which are the primary and secondary on-call-duties at this clinic.

#### Frequency of Interruptions

The frequency of interruptions from mobile devices during observation time at this clinic were considered as “normal” most of the observation days, except for one day observing a chief physician, Phys-F, at the outpatient clinic who (for a period) worked there only once a week: “It will probably be quiet on the phone today, I’m seldom here, so it depends if anybody knows I’m here”. The other days were more normal, like another chief physician, Phys-I, illustrated: “This is an average day regarding the number of calls. You know we pediatricians are so ‘kind’ and therefore easy to call/contact…”. Table 2 shows all interruptions from mobile devices received by the participating physicians at this clinic. However, some of the days had periods that were rather busy, which was illustrated by an assistant physician, Phys-H, when he announced in frustration after a phone call and an incoming page: “If somebody tries to call me on the phone, and it is busy, they page me, why? I’m busy on the phone and cannot return the call immediately.” Another assistant physician, Phys-G, also expressed her frustration after several interruptions: “I really want a system that could separate the important/critical calls from those that could wait.”

**Table 2:** Overview of total amount of interruptions from mobile devices at the ENT clinic during observation time (only interruptions from the followed physicians’ devices, except for *g*, which was a nurse’s phone, brought to the physician).^a^

			Assistant physician	Chief physician
**Preparatory/complementary work**				
		Answered	2a, 2b	7a
		Ignored		
**Outpatient ward**				
	No patient			
		Answered		5a
		Ignored		
	With patient			
		Answered		1a, 1d
		Ignored		1a
**Maternity/intensive/labor ward**				
	No patient			
		Answered	2a,2b,1f	3a, 1d
		Ignored		
	With patient			
		Answered		6a, 1b, 1g
		Ignored	1b	1a, 1g
**Surgical theatre**				
		Answered		1a
		Ignored		
**Meeting**				
		Answered		6a, 1d
		Ignored		
**Conversation room**				
		Answered	1a	
		Ignored		1a, 1d
**Other situations**				
		Answered	6a,2b,1f	17a, 3d
		Ignored	1b, 1d	

^a^a=wireless IP-phone; b=on-call-duty pager; d=private GSM; f=personal pager; g=other

As shown in Table 2, the physicians at CY also ignored incoming pages/calls, but the ignored pages/calls were not actually completely ignored. They were either answered and the caller was told that they would call back, or they hurried with the examination and the call was ignored until they finished or had a natural break in the examination.

#### Clinic Settings

The setting in the CY was slightly different than at ENT. The pagers were used less, and role or function was seldom used when they contacted each other. An assistant physician, Phys-G, pointed out: “Nobody uses role or function. When they contact each other, they call or page you personally”, and:

It is only an old habit if somebody still uses the pager to contact you while you are on on-call duty. A wireless phone should be enough to carry; actually I hate the sound from the pager, I have turned it off and I’m only using the vibration, and we don’t carry the GSM-based on-call duty phone unless we’re leaving the hospital.

The observer found that the on-call duty pager was in use also at this clinic, but not as much as wireless phones. It was observed only once that a chief physician was paged during observation time, referring to a critical situation that the chief physician had to attend immediately.

#### Wireless Phones vs Pagers

The physicians at this clinic also stated their satisfaction in carrying a wireless phone, and the majority of physicians also argued that the phone is not more interruptive than the pager. A chief physician, Phys-K, said: “The fact that we are carrying a wireless phone these days is brilliant compared to previously when we only had pagers. We are here to work and are supposed to be available within the working hours, and it is also easier to reach the other health care workers”. When questioned by the observer on interruptions from today’s phones compared with yesterday’s pagers, he answered:

With a phone in our pocket, you do not need to search for a phone whenever you need to call or answer a call. Another point is that I think there are fewer interruptions compared with when we only had pagers. Actually I think that pagers were slightly more interruptive since we had to search for a phone every time we wanted to return a call.

However, not all the physicians at this hospital were too happy about being equipped with a wireless phone, and some of them refused to carry one and would use only pagers. The observer met an older surgeon several times who did not carry a phone. This was commented on by one of the chief physicians, Phys-F:

Not everybody carries a phone even though they should. They think that there is a higher threshold to page than just to call? But they also need to locate a phone and that takes a lot of their time. There is not a phone on every corner anymore. These people have not given the new phone system time; they do not want changes and only want to keep the old pager system.

The observer discovered that this surgeon was paged several times when we met, and then borrowed the wireless phone from one of the other physicians/nurses nearby and in doing so also disturbed another health care worker. Another chief physician, Phys-K, announced after such an incident: “I am not too happy with those who “hide” away…”.

## Discussion

The purpose of this study was to learn about a physician’s workday, focusing on wireless communication, and to identify the potential to reduce unnecessary interruptions from mobile devices: in which situations, what context, and which location they should not be interrupted. Our aim was also to generally understand the health care workers’ communication pattern. Based on this study, but also previous studies done by members of our project group [13,42], we argue that there is strong evidence that interruptions from mobile devices represent a problem in hospitals and that a solution to reduce such interruptions is needed. Another important point, which some of the participants in this study address, is that they may have experienced fewer interruptions from wireless phones compared to pagers, once people has been used to carrying a phone. This issue has not been mentioned in previous studies and contrasts a finding in Sweden where widespread use of phones seemed to cause people to contact each other more often [43]. It suggests that, although interruptions might be a problem when using wireless phones, cultural shifts that develop over time in order to handle these interruptions might be able to effectively reduce the problem at some hospitals. An additional point made by one of the chief physicians, Phys-K, at CY, was that he thought a pager was slightly more interruptive since they had to locate a phone, even interrupt somebody else to borrow a phone, every time they wanted to return a page. This is related to the concern about an increased level of interruptions after introduction of wireless phones to all health care workers at a clinic. Some of the physicians in [13] expected an increased interruptions rate if wireless phones were introduced to all health care workers in the department.

The results from our study are used as input in an ongoing project [42,44,45] on designing an interruption management system to reduce some of the unnecessary interruptions a physician experiences throughout the day, and especially in situations where they should not be disturbed. Examples of such situations are: in surgery, during patient examinations, or having high-importance level conversations with patients or relatives.

### Strengths and Weakness of the Study

At the ENT clinic, we experienced quiet days with few interruptions from mobile devices. On one hand, this could be explained by the Hawthorn effect [46], but on the other hand, most likely not, since this effect reduces over time; our study lasted for almost 2 months and this did not change during our observations. That is, most of the clinics’ workers were aware of the project and that the observer was there to record interruptions from mobile devices and therefore may have been more careful in interrupting by calling or paging the physicians. We also observed that the nurses and other health care workers often sought each other out in-person and could therefore be aware of the context before they interrupted. During discussions, we became aware that this was not unusual and that it probably did not happen more frequently during our observations. The fact that the time we spent at the clinic was considered “quiet” by the health care workers could be explained by findings from earlier research regarding health care workers’ inaccuracy when reporting incidents [47,48], which also could include interruptions. But since it also was considered “quiet” regarding patient consultations, it strengthens our belief that this was representative of the normal situation. Therefore, we do not consider the result from the study to be significantly influenced by our presence at the clinic or by health care workers’ behaving differently. However, the fact that the observer dressed like a physician and was presented as a researcher made him accepted as “one of them”, which seemed vital to their communication pattern and hopefully strengthened the study.

### Findings That Conflict With Earlier Studies

The physicians in [13,42] were concerned about increased interruptions if they carried a wireless phone instead of a pager and that a phone call interrupts more than a page. When we asked the physicians we met at St. Olavs Hospital if they thought the phone was more interruptive than the pager after the introduction of wireless IP-phones, some of them told us that in the beginning, after providing wireless phones to every worker at the clinic, this might be the case. However, they said that after a while it would go back to the same level of interruptions, or even fewer interruptions with the phone. Their explanation was that it was more unpleasant to call someone and interrupt their work, than just page them. Even though the participating physicians normally answered the phone and said that they would call back, they thought that a page was more interruptive than a phone call. When they were paged, they had to locate a phone, which could result in interrupting others to borrow a phone to return the call, since they never knew how important the page was and therefore felt that they had to return it right away. With a wireless phone of their own, they could just pause what they were doing, answer the phone, sense if they had to answer right away, or just say that they would return the call, and finish up what they were doing before the interruption. This interpretation from the participants could be related to the fact that wired phones were not located “on every corner” at this hospital, since everybody was supposed to carry a wireless phone, and by borrowing a phone, they felt that they were disturbing others. A solution could be a combination of a pager for incoming calls and a wireless phone for outgoing calls like some of the physicians in [13] used. However, this solution would not solve the problem of knowing who is calling or the importance of the call. Another important consideration to have in mind about interruptions by phones vs pagers, is that since the subjects of the study had carried their own wireless phone for up to 3 years, it could be related to inaccurate reporting and retention and that the users got more used to the phone compared to the pager.

The display on top of the pager seemed to be an important feature for the physicians at the University Hospital of North Norway [13]. This display made it easier for them to see who was paging them. The physicians in this study, at St. Olavs Hospital, did not think this was important. The feedback was that such a display could be useful but not critical for the overall usability, and it is difficult to know anything about the importance of the call just from a number. We also discovered that only a few of the physicians who carried a phone and a pager, did carry the pager in a way where it could be easy to read the display.

### Summary and Future Work

We conclude from this study, but also from earlier studies [13,42], that physicians in hospitals are interrupted unnecessarily by mobile devices in situations where such interruptions should be avoided. The introduction of IP-based phones at St Olavs Hospital has shown that this transition in itself is not sufficient to reduce the number of interruptions for the physicians. The study illustrates the need for an integrated context-sensitive phone system that reduces unnecessary interruptions and eliminates the use of multiple communication devices.

We believe, by knowing and understanding the physicians’ working conditions and the nature of such interruptions and also by involving the physicians in the design process, it is possible to make a system suited for their communication pattern and working conditions. The lack of user involvement is an important issue to consider when designing and developing eHealth applications [49]. This study contributes to such knowledge and is used as input in an ongoing project on designing and developing a context-sensitive communication system: CallMeSmart. Context sensing when developing mobile medical applications has also been a success within other studies of medical Internet research [50]. Our system is designed to reduce unnecessary interruptions from mobile devices in situations where interruptions should be avoided, such as, when they are in surgery dressed in sterile clothing, during patient examination in outpatient clinic, having high-importance level conversations with patients/relatives. It is also designed to eliminate the need of multiple communication devices for each user. The system is supposed to sense the context of each user automatically, change the physicians’ availability and the phones’ profile according to the context information, and also give the caller feedback about the physicians’ availability. As such, we are developing a prototype for a context-sensitive mobile communication system suitable for hospitals, called CallMeSmart. CallMeSmart is being developed using input from this study in combination with outcomes from [42,44,45], and we are involving real users in the design and testing process.

## Abbreviations

CY: Child and Youth
ENT: Ear, Nose, and Throat
GSM: Global System for Mobile (communications)
IP: Internet Protocol
